# A New Quantitative Approach for Correcting Cirrhosis-Associated Hyponatremia by Inducing Negative Water Balance in Excess of Negative Sodium and Potassium Balance

**DOI:** 10.7759/cureus.77334

**Published:** 2025-01-12

**Authors:** Minhtri K Nguyen, Minh-Kevin Nguyen, Dhiresh Bandaru

**Affiliations:** 1 Internal Medicine and Nephrology, Ronald Reagan University of California Los Angeles Medical Center, Los Angeles, USA; 2 Internal Medicine, Ronald Reagan University of California Los Angeles Medical Center, Los Angeles, USA

**Keywords:** cirrhosis, dysnatremia, hypervolemia, hyponatremia, sodium

## Abstract

Introduction: Hypervolemic hyponatremia due to cirrhosis is caused by an increment in total body water (TBW) in excess of an increase in total exchangeable sodium (Na^+^) and potassium (K^+^). Therefore, therapy is aimed at treating not only the hyponatremia but there is an additional requirement to treat the volume overload.

Methods: Correction of cirrhosis-associated hyponatremia can be achieved by ensuring that the negative water (H_2_O) balance is in excess of the negative Na^+^ and K^+^ balance. This therapeutic approach can be attained by administering intravenous 3% sodium chloride (NaCl) and furosemide.

Results: Presently, there is no quantitative method for predicting the volume of IV 3% NaCl required to be infused in conjunction with furosemide that satisfies this therapeutic goal. Therefore, based on the empirical relationship between the plasma Na^+^ concentration and exchangeable Na^+^, K^+^, and TBW, a new formula is derived to calculate the volume of IV 3% NaCl required to raise the plasma Na^+^ concentration ([Na^+^]_p1_) to a targeted level ([Na^+^]_p2_) by attaining the desired amount of negative Na^+^, K^+,^ and H_2_O balance.

Conclusion: This new equation is the first quantitative approach for treating hypervolemic hyponatremia by attaining a negative H_2_O balance in excess of negative Na^+^ and K^+^ balance. This formula is particularly useful in the treatment of cirrhosis-associated hyponatremia where there are limited therapeutic options.

## Introduction

Hyponatremia is a common electrolyte disorder in hospitalized patients [1]. Hypovolemic hyponatremia is a disorder of negative Na^+^ and K^+^ balance in excess of negative H_2_O balance [2]. Therefore, correction of hypovolemic hyponatremia is aimed at repletion of the negative Na^+^, K^+,^ and H_2_O balance with isotonic saline. In contrast, euvolemic hyponatremia is a disorder characterized by excess total body water (TBW) and relatively neutral Na^+^ and K^+^ balance, whereas hypervolemic hyponatremia is caused by an increment in TBW in excess of an increase in total exchangeable Na^+^ and K^+ ^[2]. Hence, treatment of euvolemic hyponatremia is targeted at achieving negative H_2_O balance while maintaining neutral Na^+^ and K^+^ balance, whereas hypervolemic hyponatremia is treated by inducing negative mass balance of H_2_O (V_MB_) in excess of negative mass balance of Na^+^ and K^+^ (E_MB_). Toward this goal, intravenous 3% NaCl and furosemide can be utilized to correct hypervolemic hyponatremia. In this article, a new formula is derived to guide the treatment of hypervolemic hyponatremia by targeting negative H_2_O balance in excess of negative Na^+^ and K^+^ balance. This new formula provides the clinician with a quantitative approach to the treatment of cirrhosis-associated hyponatremia where there are limited therapeutic options.

## Materials and methods

Correction of cirrhosis-associated hyponatremia can be attained by ensuring that the negative H_2_O balance is in excess of the negative Na^+^ and K^+^ balance. Based on the study by Edelman et al., the plasma water sodium concentration ([Na^+^]_pw_) is defined by the ratio of the total exchangeable sodium (Na_e_), total exchangeable potassium (K_e_), and TBW by the following equation: [Na^+^]_pw_ = 1.11 (Na_e_ + K_e_) / TBW − 25.6 [3]. Importantly, the Edelman equation is a linear regression equation that is derived based on experimental data involving 98 patients [3]. Therefore, alterations in the mass balance of Na^+^ and K^+^ (E_MB_) in relation to changes in the mass balance of H_2_O (V_MB_) will result in a change in the ratio (Na_e_ + K_e_) / TBW, thereby altering the plasma water sodium concentration ([Na^+^]_pw_). In this article, a new formula is derived to guide the treatment of hypervolemic hyponatremia by targeting negative H_2_O balance in excess of negative Na^+^ and K^+^ balance by administering intravenous hypertonic sodium chloride (3% NaCl) and furosemide. This new formula calculates the volume of 3% NaCl infused with furosemide required to raise the plasma sodium concentration ([Na^+^]_p1_) to a targeted level ([Na^+^]_p2_) by attaining negative H_2_O balance in excess of negative Na^+^ and K^+^ balance.

## Results

Derivation of a new equation for the treatment of hypervolemic hyponatremia by targeting negative H_2_O balance in excess of negative Na^+^ and K^+^ balance

In 1958, Edelman et al. defined the empirical relationship between the plasma water sodium concentration ([Na^+^]_pw_) and the total exchangeable sodium (Na_e_), total exchangeable potassium (K_e_), and TBW [3]. Based on the Edelman equation, Nguyen previously derived a formula for predicting changes in the plasma sodium concentration ([Na^+^]_p_) resulting from simultaneous alterations in the mass balance of Na^+^, K^+,^ and H_2_O [4]:

$\text{[Na}^+\text{]}_{p2} = \frac{\left(\text{[Na}^+\text{]}_{p1} + 23.8\right)\text{TBW}_1 + 1.03 \times E_\text{MB}}{\text{TBW}_1 + V_\text{MB}} - 23.8$ (Eq. 1)

where [Na^+^]_p1_ indicates initial plasma [Na^+^]; [Na^+^]_p2_ indicates targeted plasma [Na+]; TBW_1_ indicates initial total body water; E_MB_ indicates mass balance of Na^+^ + K^+^ in a chosen duration of time.

E_MB_ = [E]_IVF_ x V_IVF_ + [E]_input_ x V_input_ - [E]_output_ x V_output_ - E]_urine_ x V_urine_

V_MB_ indicates the mass balance of H_2_O in a chosen duration of time

V_MB_ = V_IVF_ + V_input_ - V_output_ - V_urine_

On rearranging Eq. 1,

$\text{[Na}^+\text{]}_{p2} + 23.8 = \frac{\left(\text{[Na}^+\text{]}_{p1} + 23.8\right)\text{TBW}_1 + 1.03 \times E_\text{MB}}{\text{TBW}_1 + V_\text{IVF} + V_\text{input} - V_\text{output} - V_\text{urine}}$ (Eq. 2)

where V represents the volume of a given fluid in a chosen duration of time; IVF represents intravenous fluid (infusate, i.e., 3% NaCl); input represents non-infusate input (other sources of input besides 3% NaCl); output represents non-renal output (other sources of output besides urine).

Previously, Nguyen derived an equation for the correction of hypervolemic hypernatremia by inducing negative Na^+^ and K^+^ balance in excess of negative H_2_O balance [5]. In contrast, treatment of hypervolemic hyponatremia can be achieved by inducing a negative H_2_O balance in excess of negative Na^+^ and K^+^ balance. This therapeutic approach can be attained by administering intravenous 3% NaCl and furosemide. Since IV 3% NaCl is the infusate needed to treat hypervolemic hyponatremia, [E]_IVF_ = 513 mmol/L and

V_urine_ = ([E]_IVF_ x V_IVF_ + [E]_input_ x V_input_ - [E]_output_ x V_output _- E_MB_) / [E]_urine_ , rearranging Eq. 2:

$\text{[Na}^+\text{]}_{p2} + 23.8 = \frac{\left(\text{[Na}^+\text{]}_{p1} + 23.8\right)\text{TBW}_1 + 1.03 \times E_\text{MB}}{\text{TBW}_1 + V_\text{IVF} + V_\text{input} - V_\text{output} - \frac{\left([E]_\text{IVF} \times V_\text{IVF} + [E]_\text{input} \times V_\text{input} - [E]_\text{output} \times V_\text{output} - E_\text{MB}\right)}{[E]_\text{urine}}}$ (Eq. 3)

where [E] = [Na^+ ^+ K^+^] = sodium and potassium concentration

On rearranging Eq. 3,

$\text{TBW}_1 + V_\text{IVF} + V_\text{input} - V_\text{output} - \frac{\left([E]_\text{IVF} \times V_\text{IVF} + [E]_\text{input} \times V_\text{input} - [E]_\text{output} \times V_\text{output} - E_\text{MB}\right)}{[E]_\text{urine}} = \frac{\left(\left(\text{[Na}^+\text{]}_{p1} + 23.8\right)\text{TBW}_1 + 1.03 \times E_\text{MB}\right)}{\text{[Na}^+\text{]}_{p2} + 23.8}$ (Eq. 4)

On rearranging Eq. 4,

$V_{\text{IVF}} - \left([E]_{\text{IVF}} \times \frac{V_{\text{IVF}}}{[E]_{\text{urine}}}\right) = \frac{\left([Na^+]_{p1} + 23.8\right) \text{TBW}_1 + 1.03 \times E_{\text{MB}}}{\left([Na^+]_{p2} + 23.8\right)} - \text{TBW}_1 - V_{\text{input}} + V_{\text{output}} + \frac{\left([E]_{\text{input}} \times V_{\text{input}} - [E]_{\text{output}} \times V_{\text{output}} - E_{\text{MB}}\right)}{[E]_{\text{urine}}}$ (Eq. 4A)

On rearranging Eq. 4A,

$V_{\text{IVF}}\left(1 - \frac{[E]_{\text{IVF}}}{[E]_{\text{urine}}}\right) = \frac{\left([Na^+]_{p1} + 23.8\right) \text{TBW}_1 + 1.03 \times E_{\text{MB}}}{\left([Na^+]_{p2} + 23.8\right)} - \text{TBW}_1 - V_{\text{input}} + V_{\text{output}} + \frac{\left([E]_{\text{input}} \times V_{\text{input}} - [E]_{\text{output}} \times V_{\text{output}} - E_{\text{MB}}\right)}{[E]_{\text{urine}}}$ (Eq. 4B)

On rearranging Eq. 4B,

$V_{\text{IVF}} = \frac{\frac{\left([Na^+]_{p1} + 23.8\right) \text{TBW}_1 + 1.03 \times E_{\text{MB}}}{\left([Na^+]_{p2} + 23.8\right)} - \text{TBW}_1 - V_{\text{input}} + V_{\text{output}} + \frac{\left([E]_{\text{input}} \times V_{\text{input}} - [E]_{\text{output}} \times V_{\text{output}} - E_{\text{MB}}\right)}{[E]_{\text{urine}}}} {1 - \frac{[E]_{\text{IVF}}}{[E]_{\text{urine}}}}$ (Eq. 5)

## Discussion

Hyponatremia is a common electrolyte disorder in hospitalized patients [1]. The mechanisms underlying the generation of hyponatremia are defined by alterations in the mass balance of Na^+^ and K^+^ (E_MB_) in relation to changes in the mass balance of H_2_O (V_MB_) [2]. Hypervolemic hyponatremia is caused by an increment in TBW in excess of the increase in total exchangeable Na^+^ and K^+^, resulting in a relative free water excess [2]. Hypervolemic hyponatremia is commonly caused by congestive heart failure and cirrhosis. Given that hyponatremia due to congestive heart failure and cirrhosis is due to a relative free water excess, vasopressin 2-receptor antagonists, tolvaptan and conivaptan, respectively, can be used to treat hyponatremia in these clinical settings by inducing negative H_2_O balance [6,7]. However, tolvaptan, a selective vasopressin 2-receptor antagonist, is contraindicated in cirrhosis-associated hyponatremia due to the potential risk of hepatotoxicity [8]. Moreover, conivaptan, a non-selective V1a and V2 receptor antagonist is typically avoided in cirrhotic patients since it can cause hypotension in these patients with baseline low blood pressure, and it may increase the risk of variceal bleeding and worsen underlying hepatorenal syndrome [6].

Treatment of hypervolemic hyponatremia can be therapeutically challenging in cirrhotic patients who are refractory to free water restriction and diuretic therapy and who are not candidates for vasopressin 2-receptor antagonists. In these patients with cirrhosis-associated hyponatremia, treatment of hypervolemic hyponatremia is aimed at correcting the hyponatremia as well as inducing negative Na^+^, K^+,^ and H_2_O balance to treat the volume overload. Toward this goal, the infusion of 3% NaCl with intravenous furosemide can be effective in treating both hyponatremia and volume overload by inducing negative H_2_O balance in excess of negative Na^+^ and K^+^ balance.

Presently, there is no quantitative method for predicting the volume of IV 3% NaCl (V_IVF_) required to be infused with furosemide that meets this goal. Based on the empirical interrelationship between the plasma water [Na^+^] and exchangeable sodium (Na_e_), exchangeable potassium (K_e_), and TBW as defined by the Edelman equation: [Na^+^]_pw_ = 1.11 (Na_e_ + K_e_) / TBW − 25.6 [3], alterations in the mass balance of Na^+^ and K^+^ (E_MB_) in relation to changes in the mass balance of H_2_O (V_MB_) will result in a change in the ratio (Na_e_ +K_e_) / TBW, thereby altering the plasma water sodium concentration ([Na^+^]_pw_). Therefore, equation 5 is derived to guide the treatment of hypervolemic hyponatremia by targeting negative H_2_O balance in excess of negative Na^+^ and K^+^ balance. Equation 5 determines the volume of IV 3% NaCl (V_IVF_) required to raise the initial plasma Na^+^ concentration ([Na^+^]_p1_) to the desired level ([Na^+^]_p2_) by inducing the targeted amount of negative Na^+^ and K^+^ balance (E_MB_). Since equation 5 calculates the volume of IV 3% NaCl needed to raise the [Na^+^]_p_ as well as to achieve the targeted negative E_MB_, the derivation of this equation accounts for the fact that the negative mass balance of H_2_O (V_MB_) must be in excess of the negative mass balance of Na^+^ and K^+^ (E_MB_). Simply put, in order for the [Na^+^]_p_ to be increased in the setting of negative E_MB_, the negative V_MB_ must be greater than the negative E_MB_.

Clinical utility of equation 5

To illustrate the clinical utility of equation 5, let’s calculate the volume of IV 3% NaCl required to increase the [Na^+^]_p_ from 124 mmol/L to 130 mmol/L while attaining a targeted negative E_MB_ of -100 mmoles with furosemide in a 72 kg male with hypervolemic hyponatremia due to cirrhosis.

Parameters entered into equation 5 are: [Na^+^]_p1_ of 124 mmol/L; [Na^+^]_p2_ of 130 mmol/L; TBW_1_ of 43.2 liters; E_MB_ of -100 mmoles; [Na^+^ + K^+^]_urine_ = 80 mmol/L.

According to equation 5, 0.204 liters of IV 3% NaCl would be needed to raise the [Na^+^]_p_ from 124 mmol/L to 130 mmol/L while attaining the desired negative E_MB_ of -100 mmoles. Hence, furosemide drip was titrated to induce a total urinary output of ~2.56 liters (V_urine_ = ([E]_IVF_ x V_IVF_ + [E]_input_ x V_input_ - [E]_output_ x V_output_ - E_MB_) / [E]_urine_ = 2.56 L). Given that the [Na^+^]_p_ increased from 124 mmol/L to 130 mmol/L in the setting of negative Na^+^ + K^+^ balance, the negative mass balance of H_2_O (V_MB_) must exceed the negative mass balance of Na^+^ and K^+^ (E_MB_). The E_MB_ in this case was -100 mmol (E_MB_ = [E]_IVF_ x V_IVF_ - [E]_urine_ x V_urine_ = 513 x 0.204 - 80 x 2.56 = -100 mmol), and the V_MB_ was -2.36 L (V_MB_ = V_IVF_ - V_urine_ = 0.204 - 2.56 = -2.36 L). As a result, the net fluid loss resulting from the negative mass balance of Na^+^, K^+,^ and H_2_O was hypotonic (E_MB _/ V_MB_ = -100 mmol / -2.36 L= 42 mmol/L) to the patient’s [Na^+^]_p_, thereby resulting in an increase in the [Na^+^]_p_. This can be verified by the empirical interrelationship between the [Na^+^]_p_ and the exchangeable Na^+^ (Na_e_), exchangeable K^+^ (K_e_), and TBW [9]:

\begin{equation*} [\text{Na}^+]_p = \frac{1.03 (\text{Na}_e + \text{K}_e)}{\text{TBW}} - 23.8 \end{equation*} (Eq. 6)

Therefore,

\begin{equation*} \text{Na}_e + \text{K}_e = \frac{\left( [\text{Na}^+]_p + 23.8 \right) \times \text{TBW}}{1.03} \end{equation*} (Eq. 7) 

Na_e1_ + K_e1_ = (124 + 23.8) x 43.2 / 1.03 = 6199 mmol

Na_e2_ + K_e2_ = Na_e1_ + K_e1_ + E_MB_ = 6199 - 100 = 6099 mmol 

TBW_2_ = TBW_1_ + V_MB_ = 43.2 - 2.36 = 40.84 liters

Therefore,

\begin{equation*} [\text{Na}^+]_{p2} = \frac{1.03 (\text{Na}_{e2} + \text{K}_{e2})}{\text{TBW}_2} - 23.8 \end{equation*}

\begin{equation*} [\text{Na}^+]_{p2} = \frac{1.03 \times 6099}{40.84} - 23.8 = 130 \, \text{mmol/L} \end{equation*}

Therefore, equation 5 accurately calculates the volume of IV 3% NaCl (V_IVF_) required to raise the [Na^+^]_p_ from 124 mmol/L to 130 mmol/L in conjunction with IV furosemide in achieving negative H_2_O balance in excess of negative Na^+^ + K^+^ balance.

Aquaresis versus natriuresis

It is well known that furosemide induces a natriuresis, whereas tolvaptan induces an aquaresis, which results in a greater degree of electrolyte-free water clearance. In cirrhotic patients with hypervolemic hyponatremia in whom tolvaptan is contraindicated, the utility of IV 3% NaCl in conjunction with IV furosemide is an alternative approach to increase net electrolyte-free water clearance. As the targeted negative E_MB_ approaches zero, the net electrolyte-free water clearance increases accordingly and simulates the effect of tolvaptan. Using the clinical example above, the net electrolyte-free water clearance can be calculated at various targeted E_MB_ by using the whole-body electrolyte-free water clearance (WB-EFWC) formula [10]:

\begin{equation*} \text{WB-EFWC} = V_{\text{MB}} - \frac{1.03 \, E_{\text{MB}}}{[\text{Na}^+]_p + 23.8} \end{equation*} (Eq. 8)

where WB-EFWC indicates whole-body electrolyte-free water clearance; [Na^+^]_p_ indicates plasma Na^+^ concentration; E_MB_ indicates mass balance of Na^+^ + K^+^ = [E]_input_ x V_input_ - [E]_output_ x V_output_; V_MB_ indicates mass balance of H_2_O = V_input _- V_output_; [E] = [Na^+^ + K^+^] = Na^+^ and K^+^ concentration; V indicates volume.

Rather than considering only the renal component of electrolyte-free water clearance, this formula calculates the whole-body electrolyte-free water clearance for a given mass balance of Na^+^, K^+^, and H_2_O by taking into account all sources of input and output of Na^+^, K^+^, and H_2_O [10].

As shown in Table 1, as the negative E_MB_ decreases in value from -250 mmoles to 0 mmole, the WB-EFWC increases, resulting in a greater degree of aquaresis. When the targeted E_MB_ is zero, the aquaresis results in a more effective correction of the hyponatremia, requiring only a negative V_MB_ of -1.68 L to raise the [Na^+^]_p_ from 124 mmol/L to 130 mmol/L. On the other hand, as the negative E_MB_ increases in value from 0 mmole to -250 mmoles, the WB-EFWC decreases, resulting in a greater degree of natriuresis. When the targeted E_MB_ is -250 mmoles, the natriuresis results in less effective correction of the hyponatremia, requiring a negative V_MB_ of -3.36 L to raise the [Na^+^]_p_ from 124 mmol/L to 130 mmol/L. However, the greater natriuresis will result in greater volume losses, thereby leading to more effective treatment of the volume overload. Therefore, the desired aquaresis versus natriuresis can be targeted based on the severity of the hyponatremia and the degree of volume overload.

**Table 1:** Aquaresis versus natriuresis [Na^+^]_p_: plasma Na^+^ concentration; TBW: total body water; [Na^+^ + K^+^]_IVF_: infusate sodium and potassium concentration; [Na^+^ + K^+^]_urine_: urinary sodium and potassium concentration; V_IVF_: infusate volume; V_urine_: urinary volume; V_MB_: mass balance of H_2_O; E_MB_: mass balance of Na^+^ + K^+^; WB-EFWC: whole-body electrolyte-free water clearance

Initial [Na^+^]_p _(mmol/L)	Targeted [Na^+^]_p _(mmol/L)	Initial TBW (L)	[Na^+^+ K^+^]_IVF _(mmol/L)	[Na^+ ^+ K^+^]_urine_ (mmol/L)	V_IVF _(L)	V_urine _(L)	V_MB _(L)	E_MB_ (mmol)	WB-EFWC (L)
124	130	43.2	513	80	0.311	1.997	-1.685	0	-1.685
124	130	43.2	513	80	0.258	2.278	-2.020	-50	-1.672
124	130	43.2	513	80	0.204	2.559	-2.355	-100	-1.658
124	130	43.2	513	80	0.151	2.840	-2.690	-150	-1.645
124	130	43.2	513	80	0.097	3.122	-3.025	-200	-1.631
124	130	43.2	513	80	0.043	3.403	-3.360	-250	-1.617

Limitations of equation 5

There are several limitations inherent in Eq. 5 that need to be considered in the treatment of patients with hypervolemic hyponatremia. First, given that there may be dynamic changes in the mass balance of Na^+^, K^+^, and H_2_O during therapy, the patient’s input and output of Na^+^, K^+^, and H_2_O and the plasma [Na^+^] must be monitored closely to guide additional adjustments in the fluid prescription. The constancy of input and output sources in any given patient will determine the frequency with which this needs to be done. Last, since TBW is a variable in Eq. 5, the accuracy of Eq. 5 relies on an accurate estimate of TBW. In this context, the regression equations reported by Watson et al. can be used to obtain an accurate estimate of TBW [11].

## Conclusions

In conclusion, hypervolemic hyponatremia due to cirrhosis is caused by an increment in TBW in excess of an increase in total exchangeable Na^+^ and K^+^, resulting in a relative free water excess. Since these patients are hypervolemic and hyponatremic, therapy is aimed at correcting not only the hyponatremia but also attaining negative Na^+^, K^+,^ and H_2_O balance. Therefore, correction of hypervolemic hyponatremia can be attained by administering intravenous 3% NaCl and furosemide to achieve a negative H_2_O balance in excess of negative Na^+^ and K^+^ balance. In this article, a new formula is derived to determine the volume of IV 3% NaCl (V_IVF_) that needs to be infused that fulfills these requirements. Importantly, this new equation is derived based on the Edelman equation, which is a linear regression equation that is determined based on experimental data involving 98 patients. This new formula is the first quantitative approach for treating hypervolemic hyponatremia by attaining a negative H_2_O balance in excess of negative Na^+^ and K^+^ balance. This new formula will be particularly helpful in providing the clinician with a quantitative method for the correction of cirrhosis-associated hyponatremia where there are limited therapeutic options.
